# Health financing at district level in Malawi: an analysis of the distribution of funds at two points in time

**DOI:** 10.1093/heapol/czx130

**Published:** 2017-10-27

**Authors:** Josephine Borghi, Spy Munthali, Lameck B Million, Melisa Martinez-Alvarez

**Affiliations:** 1 Department of Global Health and Development, London School of Hygiene & Tropical Medicine, 15-17 Tavistock Place, London WC1H 9SH, UK,; 2 Chancellor College, University of Malawi, Zomba, Malawi P.O. Box 280 and; 3 Malawi National Statistics Office, Malawi

**Keywords:** Sub-national, health financing, Malawi, district, equity, child health

## Abstract

There is growing attention to tracking country level resource flows to health, but limited evidence on the sub-national allocation of funds. We examined district health financing in Malawi in 2006 and 2011, and equity in the allocation of funding, together with the association between financing and under five and neonatal mortality. We explored the process for receiving and allocating different funding sources at district level. We obtained domestic and external financing data from the Integrated Financial Management Information System (2006–11) and AidData (2000–12) databases. Out-of-pocket payment data came from two rounds of integrated household budget surveys (2005; 2010). Mortality data came from the Multiple Indicator Cluster Survey (2006) and Demographic and Health Survey (2010). We described district level health funding by source, ran correlations between funding and outcomes and generated concentration curves and indices. 41 semi-structured interviews were conducted at the national level and in 10 districts with finance and health managers. Per capita spending from all sources varied substantially across districts and doubled between 2006 and 2011 from 7181 Kwacha to 15 312 Kwacha. In 2011, external funding accounted for 74% of funds, with domestic funding accounting for 19% of expenditure, and out of pocket (OOP) funding accounting for 7%. All funding sources were concentrated among wealthier districts, with OOP being the most pro-rich, followed by domestic expenditure and external funding. Districts with higher levels of domestic and external funding had lower levels of post-neonatal mortality, and those with higher levels of out-of-pocket payments had higher levels of 1–59 month mortality in 2006. There was no association between changes in financing and outcomes. Districts reported delayed receipt of lower-than-budgeted funds, forcing them to scale-down activities and rely on external funding. Governments need to track how resources are allocated sub-nationally to maximize equity and ensure allocations are commensurate to health need.

Key Messages

- There is substantial variation in levels of health funding across districts in Malawi
- Districts in Malawi are heavily reliant on external sources of funds for health, with domestic funds coming late and below levels budgeted
- Districts with higher levels of domestic and external funding had lower levels of post-neonatal mortality, and those with higher levels of out-of-pocket payments had higher levels of mortality in earlier years, but there was no association between changes in funding levels and outcomes over time.

## Introduction

There is global recognition of the importance of health sector financing for strengthening health systems to improve health outcomes (WHO Commission on Macroeconomics and Health 2001; Makuta and O’Hare 2015; Farag *et al.* 2013) including the need to generate more domestic resources for health as highlighted in the third international conference on financing and development in Addis Ababa. The question of how to generate and invest funding to maximize access to affordable and efficient care is of concern to governments everywhere, and a particular challenge for low-income countries (LICs), where the government resource base is more limited. In recognition of this, many country governments have developed health sector financing strategies, and integrated financing objectives into health sector plans. Such strategies include mechanisms to enhance fiscal space for health by improving the efficiency of tax revenue collection (Doherty *et al*. 2015) and finding ways to ensure pre-payment for health care among the informal sector through tax or insurance contributions. Many governments have removed user fees for maternal and child health services (McKinnon *et al*. 2015; Hatt *et al.* 2013), in a bid to reduce the burden of out of pocket (OOP) payments and improve access to these services, reflecting an implicit commitment to increasing public funding for these services. Global donors also continue to invest heavily in the health sector in LICs (Dieleman *et al.* 2016b).

With the increased focus on health financing, it is important to track resources to monitor progress towards addressing financing and broader health goals. Numerous studies have examined resource flows from development partners to the health sector and to specific population beneficiary groups (e.g. Dieleman *et al*. 2015a; Dieleman *et al*. 2015b; Graves *et al*. 2015; Grollman *et al*. 2017; Haakenstad *et al*. 2016; Schneider *et al*. 2016). A number of studies have also sought to examine financing trends across a range of financing sources within the African region (Kirigia *et al.* 2006; Nguyen *et al.* 2011; Sambo *et al.* 2013). There is now a growing interest in understanding the trends in health sector funding at the country level (Dieleman *et al*. 2016a), including domestic and external funding and how these relate to OOP payments and the affordability of care. A number of studies have examined trends in total health expenditure by source at country level drawing from national health accounts data in Nigeria (Lawanson *et al.* 2012), in China (Long *et al.* 2013), in Iran (Zakeri *et al.* 2015) and in Malawi, (Zere *et al.* 2010). Others have examined financial flows to specific health areas, such as reproductive, maternal, newborn and child health (RMNCH) at country level in Kenya (Sidze *et al.* 2013), Namibia (Mbeeli *et al.* 2011) and Burundi (Chaumont *et al.* 2015). However, making progress is contingent not only on generating sufficient resources at the national level, but also on funds being equitably allocated across sub-national areas (regions/districts) (Ensor *et al.* 2012). Yet there is much less evidence of the availability of health sector funding at the sub-national level in countries. To our knowledge only three papers have examined sub-national funding levels, across China (Brixi *et al.* 2013), Peru and Pakistan (Lorenz and Khalid 2011), and one province of Iran (Mehrolhassani *et al.* 2014). However, no studies were identified in Sub-Saharan Africa, and none considered how funding changed over time.

This study adds to existing evidence by examining the levels of funding and sources of financing for health in Malawi at the district-level at two points in time: 2006 and 2011 and exploring the distribution of financing in relation to wealth, and the association between funding levels and neonatal, post-neonatal and under five mortality. Malawi was chosen as a case due to the substantial reductions in child mortality achieved over the period 2000–13, coupled with increased service coverage (Kanyuka *et al.* 2016). This study was carried out as part of the Countdown project, a multi-disciplinary multi-institutional initiative tracking progress towards RMNCH globally and at the country level, focusing on understanding the coverage, health system and financial factors explaining mortality improvements in countries.

## Study setting

### Health financing

Total health expenditure in Malawi over the period 2006–11 grew almost 3-fold from 47 to 129 billion Kwacha, though the share of the domestic budget allocated to health has been consistently below the 15% recommended in the Abuja declaration (World Health Organization 2011), estimated at 7.2% in 2011 (Kanyuka *et al.* 2016). Donor funding represented 66-70% of total health expenditure, with a fairly constant share of OOP payments at around 10% (Kanyuka *et al.* 2016). Funding to priority areas has grown substantially, e.g. there was a 6.3-fold increase in total funding to child health between 2006 and 2011 and a 3.6-fold increase in funding to maternal and newborn health and family planning, which is in large part driven by an increase in external funding to these areas from $31.2 m in 2003 to $102.2 m in 2012 for child health; and from $16.5 m in 2003 to $42.6 m in 2012 for maternal and newborn health (Kanyuka *et al.* 2016).

**Figure 1.**
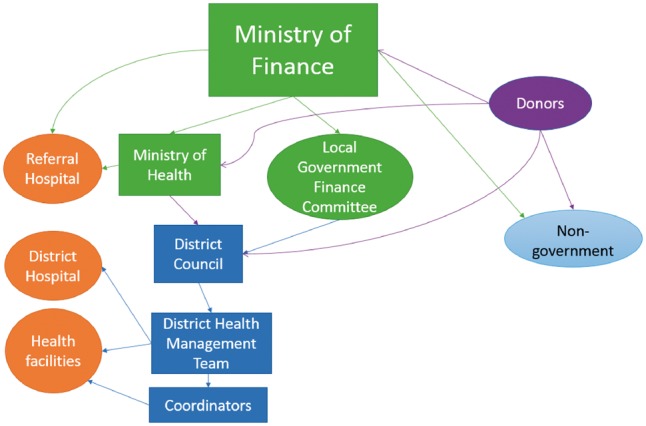
Schematic representation of the flow of funds in the Malawian health sector. Note to figure: arrows represent (financial) resource flows

Health care financing and management in Malawi is decentralized. At the district level, health care is managed by a District Health Management Team (DHMT). Districts develop the District Implementation Plan (DIP), annual plans for health service delivery and related budgets, in consultation with providers and communities. Districts receive funds from the Ministry of Finance (MoF) as block grants to cover district level health activities. These funds originate from domestic sources (tax revenue) and from external sources, through general budget support. Districts also received basket funding from donors, pooled funds for the health sector as part of the Sector Wide Approach (SWAP), since 2004. These funds are channelled to districts through the Ministry of Health (MoH). Donor funds can also be channelled straight to districts through the Local Government Financing Committee^1^ or through non-government channels as vertical (discrete) programmes (Figure 1). Health services at government facilities and at selected non-profit private facilities contracted by the MoH are officially free at the point of use (Chirwa *et al.* 2013). However, in practice, if drugs are out of stock then patients will pay OOP at private pharmacies. Patients also pay for care in private for profit facilities. OOP payments are not a source of financing for districts in Malawi, nor would levels of OOP payments be taken into account within resource allocation decisions.

### Child health

We focus on the relationship between financing and child health outcomes as reductions in under five mortality in Malawi have been substantial between 1990 and 2013, from 247 deaths (90% CI 234–262) per 1000 livebirths to 71 deaths (58–83) in 2013, representing an annual decline of 5.4% (Kanyuka *et al.* 2016). Much of the decline was in the 1–59 months age group, with neonatal mortality declining at a slower rate (from 50 to 23 deaths per 1000 livebirths), therefore, we also consider the relationship between financing and neonatal mortality. District level financing primarily benefits primary care, of which pregnant women and children under five are substantial beneficiaries.

## Methods

### Time frame

We focus on the years 2006 and 2011, to examine financing in Malawi during the period after the introduction of the SWAP. These are also the 2 years for which complete data were available from all financing sources, and with available mortality data from household surveys, enabling a comparison to be made between levels of financing and health-related outcomes. However, it is important to note that financing data is not available for consistent time periods, as explained below, and in some cases the figures provided are estimates or proxies for the years of interest. In our results we refer consistently to years 2006 and 2011.

### Data sources

#### Domestic expenditure

Approved district budget and revised budget figures from the Integrated Financial Management Information System (IFMS) system for financial years 2005/06 and 2010/11 were used as the closest proxies for domestic expenditures at district level for years 2006 and 2011. In addition to central level domestic funding this also includes externally sourced general budget support which is channelled through the MoF as well as locally collected revenues that do not go back to the central government level.

#### External expenditure

Data on external expenditure on health were obtained from the AidData database (Peratsakis 2012), which provides information on donor funding for over 80% of all donors, reporting total donor disbursements per project, together with the years for which a project is active, for each district. Data include funding through pooled funding mechanisms such as the sector wide approach and general budget support, however, in practice the latter funding were not present within the database. The database provides information for each district, on project expenditures across all sectors. Expenditure figures are provided as totals for projects for the life time of the project. Information is also available on the duration of the project (start and end years). To calculate disbursements per district for the years 2006 and 2011, we restricted our analysis to those projects which were active in either of these years and were classified as being made to the health sector, and divided the total disbursement amount for a given project by the total number of years that the project was active to estimate annual disbursements. Records were identified within the health sector. Disbursements with information missing for a given year were not included.

#### Out-of-pocket payments

We derived data on annual OOP payments from two rounds of the Integrated Household Survey data that were closest to the years of focus in this study (2005 and 2010), a nationally representative survey of income and expenditure. These figures were taken as proxies for OOP payments in 2006 and 2011. The survey records OOP payments for the most recent outpatient visit, as well as the number of outpatient visits in the previous 4 weeks for every member in the household. Total OOP payments were estimated as the sum of total outpatient and inpatient expenditures for each household. For each household member, we multiplied reported OOP payments for the most recent outpatient visit by the number of visits reported in the past 4 weeks, and multiplied this by 12 to estimate annual household expenditures on outpatient care. The survey also asks respondents how many inpatient admissions they experienced in the previous 12 months, and OOP expenditure associated with the last admission. We multiplied the number of admissions by the costs per admission to estimate annual household inpatient admission expenditures. We estimated average annual costs per capita by dividing total household costs by the number of household members. We estimated total OOP expenditures per district by multiplying the average household cost per district by the estimated number of households in each district.

#### Population, socio-economic status and health outcomes

Population statistics by district were obtained from projections based on the 2008 census data produced by the National Statistics Office (National Statistics Office 2008). We used population data for 2006 and 2011 to estimate per capita figures. Data on annual household consumption expenditure were obtained from the Integrated Household Surveys for 2005 and 2010, and per capita estimates were generated by dividing these figures by household size. Data on neonatal, post-neonatal (1–59 months) and under five mortality were based on the Multiple Indicator Cluster Survey data (2006) and Demographic and Health Survey (DHS) data (2010) as the closest time point available to 2011, using methods described previously (Kanyuka *et al.* 2016).

The district level analysis excludes the district of Likoma because of its small population size and combined Mwanza and Neno district, as Neno district was formed as a separate district after 2006. In the analysis of household surveys, urban and rural households were combined at district level (e.g. Blantyre city was combined with Blantyre district, Kanyuka *et al.* 2016).

### Qualitative data

We conducted 41 semi-structured key informant interviews with government stakeholders at central and district levels to determine how they allocate and prioritize funding across districts for health, and to identify potential problems and bottlenecks in resource flows (Table 1). District-level interviews were conducted in ten districts, representing all three regions, and with mixed performance in terms of child health outcomes. Stakeholders were sampled purposively; snowballing was used to identify further relevant stakeholders. Interviews were recorded and transcribed by a professional transcriptionist in all but two cases where consent was not granted (notes were taken instead).

**Table 1.** Stakeholders interviewed

Stakeholder	Number
Ministry of Finance	1
Ministry of Health	2
District Council Director of Finance	9
District Health Officer/District Medical Officer/District Nursing Officer	9
District Health Accountant	8
District Reproductive Health coordinator/Safe Motherhood Coordinator	9

### Data analysis

We estimated per capita district health expenditures by source for 2006 and for 2011 for each of the 26 districts of Malawi, both in real terms and as a share of total per capita funding for each district. We illustrated findings for each year using bar charts and examined changes in funding levels over time for each funding source. Pairwise correlation analyses were performed to assess the relationship between district per capita health financing levels by source and district level outcomes (neonatal mortality, under five mortality and post-neonatal mortality) in 2005/06 and in 2010/11 to examine the relationship between changes in financing levels and changes in outcomes between these two time periods. The pairwise correlation coefficient and *P*-value for the correlation coefficient are presented.

To examine the distribution of health sector funding in relation to district socio-economic status we estimated concentration indices for each financing source, and concentration and Lorenz curves. We compared the distribution of funding, depicted by the concentration curve, against the 45 degree line of perfect equality. If the concentration curve lies above (below) the 45 degree line, the distribution is pro-poor (pro-rich). A positive concentration index indicates a pro-rich distribution and a negative index a pro-poor distribution, with values lying between 0 and 1. Heat maps were generated to represent data on health expenditure by source and child mortality graphically using the ArcGIS software. All costs were inflated to 2013 Kwacha prices using average annual inflation rates, and are presented in 2013 constant prices. AidData disbursements are reported in US dollars. They were inflated into 2013 prices based on annual US inflation rates, and converted into Kwacha using the average 2013 exchange rate (1 USD = 369 Kwacha).

Interview data were analysed using thematic content analysis. The themes were driven by the interview guide, although open coding was undertaken on a sample of four transcripts to identify any new themes emerging. Themes centred around decisions on budget guidelines, allocation of health resources, and changes over time in financing procedures and resource flows.

## Results

**Figure 2.**
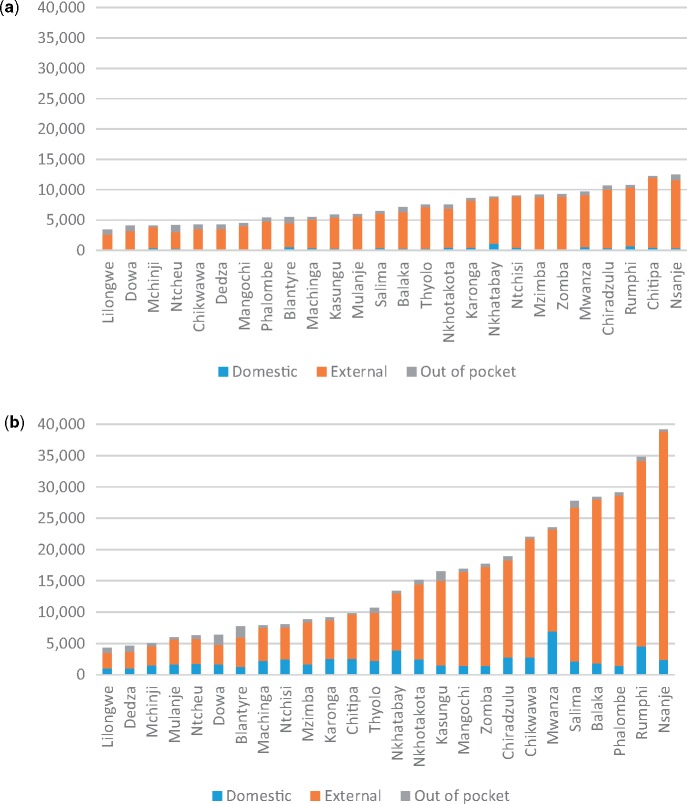
(a) District health expenditure per capita by source in 2006 (2013 Kwacha). (b) District health expenditure per capita by source in 2011 (2013 Kwacha)

All districts received funds from government, external sources and OOP payments by individuals in 2006 and in 2011. However, there were wide disparities between districts, both in the total per capita health expenditures and the levels of expenditure by source (Figure 2a and b). There was also a substantial change in the levels of total health expenditure per capita over the period 2006–11. Average total health expenditure per capita at district level increased from 7181 Kwacha to 15 312 Kwacha between 2006 and 2011 (more than doubling in real terms). Total health expenditure per capita varied from 3449 Kwacha in Lilongwe district to 12 486 Kwacha in Nsanje in 2006; and from 4300 Kwacha in Lilongwe district to 39 149 Kwacha in Nsanje in 2011. There was an increase in total health expenditure in 22 out of 26 districts between 2006 and 2011 by an average of 142%, and a decline by an average of 9% in the remaining 4 districts.

Domestic expenditure per capita increased from an average of 390 Kwacha per capita across all districts to 2284 Kwacha (a 6-fold increase) over the period. The increase in domestic funding was noted in every district, with an average relative increase of 82%. The highest level of domestic funding per capita was in Nkhata Bay in 2006 (1129 Kwacha), and in Mwanza in 2011 (6910 Kwacha), and the lowest level in Lilongwe in 2006 (166 Kwacha) and Dedza in 2011 (1073 Kwacha).

External funding per capita increased from 6217 Kwacha per capita across all districts to 12 377 Kwacha between 2006 and 2011, almost doubling over this period. There was an increase in external funding in 18 districts and a reduction in 8, with an average relative increase of 157%. There was substantial variation in external expenditure levels (ranging from 2452 Kwacha in Lilongwe to 11 498 in Chitipa in 2006; and from 2446 Kwacha in Lilongwe to 36 590 Kwacha in Nsanje in 2011). External expenditure levels were also substantially larger than domestic health expenditure levels in all districts though the relative difference in magnitude reduced over time. External funding levels ranged from seven times higher than domestic funding in Nkhata Bay (2006) to 54 times higher in Zomba in the same year (the average ratio of external to domestic funding across districts was 18). In 2011, the ratio of external to domestic funding went from just under two in Dowa to 15 in Nsanje (average ratio of six).

OOP expenditure increased minimally from an average of 574 Kwacha per capita to 651 Kwacha between 2006 and 2011, varying from 155 Kwacha in Ntchisi to 1130 Kwacha in Ntcheu in 2006; and from 161 Kwacha in Chitipa to 1712 Kwacha in Blantyre in 2011. OOP expenditures per capita increased in 16 districts by 5% and reduced in 10 over this period by 5%.

The financing mix has evolved substantially between 2006 and 2011, with domestic funding accounting for 6% of total health expenditure in 2006 increasing to 19% in 2011; and external funding reducing from 85 to 74% and the share of OOP payments reducing from 10 to 7%. In both years, external expenditure was the biggest source of district level funding making up >¾ of total health expenditure (Table 2).

**Table 2.** Health expenditure by source as a proportion of total health expenditure by district in the financial years 2006 and 2011

District	2006			2011		
	Domestic per capita (% total)	External per capita (% total)	OOP per capita (% total)	Domestic per capita (% total)	External per capita (% total)	OOP per capita (% total)
Balaka	4.5%	83.9%	11.6%	6.5%	92.1%	1.4%
Blantyre	9.9%	72.6%	17.5%	16.5%	61.3%	22.2%
Chikwawa	6.1%	76.6%	17.3%	12.5%	85.9%	1.6%
Chiradzulu	3.9%	90.0%	6.0%	14.7%	81.9%	3.4%
Chitipa	4.2%	94.0%	1.8%	26.1%	72.2%	1.6%
Dedza	5.7%	76.7%	17.6%	23.3%	55.7%	20.9%
Dowa	6.1%	72.6%	21.2%	26.2%	49.3%	24.5%
Karonga	5.5%	89.7%	4.7%	28.0%	67.0%	5.0%
Kasungu	5.3%	86.2%	8.5%	9.3%	81.6%	9.1%
Lilongwe	4.8%	71.1%	24.1%	25.0%	56.9%	18.1%
Machinga	6.7%	86.3%	7.0%	28.3%	66.2%	5.5%
Mangochi	5.0%	82.5%	12.5%	8.4%	88.6%	3.0%
Mchinji	8.6%	84.1%	7.3%	29.7%	61.2%	9.1%
Mulanje	4.4%	87.9%	7.6%	27.6%	66.2%	6.2%
Mwanza	5.9%	87.6%	6.5%	29.4%	69.3%	1.3%
Mzimba	2.8%	92.4%	4.8%	19.0%	75.7%	5.3%
Nkhatabay	12.7%	84.5%	2.8%	29.2%	67.5%	3.4%
Nkhotakota	6.7%	84.6%	8.7%	16.3%	78.9%	4.7%
Nsanje	3.4%	89.4%	7.2%	6.1%	93.2%	0.7%
Ntcheu	7.0%	65.7%	27.3%	28.1%	62.9%	8.9%
Ntchisi	4.9%	93.4%	1.7%	30.3%	63.8%	5.9%
Phalombe	3.6%	84.1%	12.3%	5.0%	93.6%	1.5%
Rumphi	6.8%	89.4%	3.8%	13.1%	85.1%	1.8%
Salima	6.4%	87.2%	6.4%	7.8%	88.7%	3.5%
Thyolo	3.7%	91.0%	5.3%	20.6%	71.5%	7.9%
Zomba	1.7%	93.8%	4.5%	8.2%	89.0%	2.8%
District average	5.6%	84.5%	9.8%	19.1%	74.0%	6.9%

Note to table: these figures have been rounded to the nearest decimal; hence the rounded figures may not always add up to 100.

**Figure 3.**
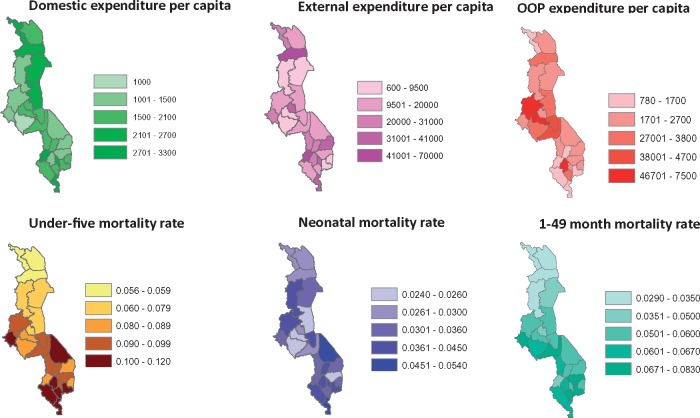
Heat maps showing the district distribution of health expenditure per capita by source (Kwacha) and child mortality rate indicators

In 2006, higher levels of domestic and external expenditure per capita were associated with significantly lower levels of post-neonatal mortality (Table 3; Figure 3), with a borderline significant negative association also found between domestic funding and under five mortality. A positive association was found between external funding and neonatal mortality, which was significant at *P* < 0.1. Districts with higher levels of OOP expenditure had significantly higher levels of under-five mortality and post-neonatal mortality. In 2011 the relationship between domestic funding and under five and post-neonatal mortality was slightly stronger. For external funding the positive association with neonatal mortality was maintained. In 2011, there was no longer a significant association between OOP and mortality. There was no association between the changes in financing indicators between 2006 and 2011 and changes in mortality rates.

**Table 3.** Comparing associations between funding levels and newborn and child health outcomes in 2006 and 2011

		Neonatal mortality	Under five mortality	Mortality between 1 and 59 months
Domestic health expenditure per capita	2006 Corr (*P*-value)	–0.057 *(0.783)*	–0.38*(0.055)	–0.41**(0.037)
	2011 Corr (*P*-value)	–0.04 *(0.815)*	–0.39*(0.052)	–0.44**(0.025)
External health expenditure per capita	2006 Corr (*P*-value)	0.34*(0.094)	–0.22 *(0.279)*	–0.40**(0.041)
	2011 Corr (*P*-value)	0.38*(0.054)	0.03 *(0.886)*	–0.17 *(0.395)*
OOP per capita	2006 Corr (*P*-value)	–0.04 *(0.838)*	0.41**(0.040)	0.48**(0.012)
	2011 Corr (*P*-value)	–0.15 *(0.458)*	0.14 *(0.501)*	0.25 *(0.220)*

* *P* < 0.1;

** *P* < 0.05;

*** *P* < 0.01.

**Figure 4.**
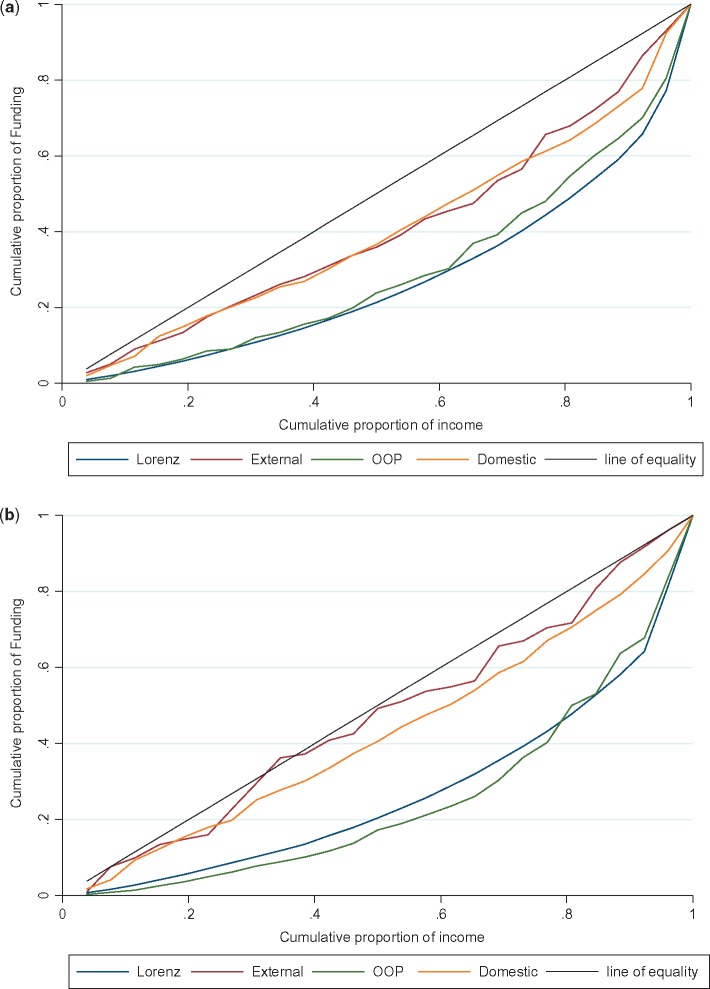
(a) Lorenz and Concentration curves for health sector funding 2006. This graph illustrates the concentration curves for each source of financing, together with the Lorenz curve for income, and the 45 degree line. If the concentration curve lies above (below) the 45 degree line, the distribution is pro-poor (pro-rich). (b) Lorenz and Concentration curves for health sector funding 2011. This graph illustrates the concentration curves for each source of financing, together with the Lorenz curve for income, and the 45 degree line. If the concentration curve lies above (below) the 45 degree line, the distribution is pro-poor (pro-rich)

In both years the distribution of OOP was the most pro-rich of the three financing sources, followed by domestic funding, with external funding being the least pro-rich (Table 4, Figure 4a and b). Although OOP became more concentrated among wealthier districts between the two time periods, the distribution of external funding moved from pro-rich to proportional, and the concentration of domestic funding among wealthier districts reduced somewhat.

**Table 4.** Concentration indices for each source of financing by year

Equity measure	2006	2011
Domestic expenditure	0.20***	0.15***
External expenditure	0.19***	0.06
OOP payments	0.41***	0.50***
Income (Gini coefficient)	0.45	0.46

*** Statistical significance of the concentration index, *P* < 0.001.

### Qualitative findings

Interviews with various government representatives revealed a number of issues with domestic and external funds.

Respondents highlighted a number of challenges in relation to the receipt of government funds. First, government funds were universally perceived to be insufficient relative to needs, with the budget reportedly remaining constant despite inflation.

*[…] the budget eventually gives problem[s] when the prices of commodities have gone up. For example if you have a budget of maybe a rim of paper at 2000 in June you shouldn’t expect that 2000 to be there in October, normally the prices never go down they go up […]* — District level respondent

In addition, the amounts received were generally lower than the amounts that had been budgeted, with the shortfall varying between 5–10 and 90%, especially during the second half of the financial year.

*[…] last year we would get 10% of our monthly funding maybe instead of getting 2 million we would get 200 thousand, which is not even enough to pay our utility bills. So it brought the council in a deficit position to pay utility bills and other things […].* — District level respondent

Second, respondents reported delays in the receipt of funds, particularly at the beginning of the financial year. Delays were caused at the central level by districts not submitting their reports on time, and the erratic behaviour of donors due to the ‘ministry is not meeting the conditionalities or the ministry is not accounting for the resources’ (Ministry of Finance representative), and at the district level due to having a single pooled account for all sectors, as if the person managing the account (director of finance) was away, the release of funds would be delayed.

Delays in receiving less-than-budgeted funds resulted in the scale-down of health-related activities. For instance, one respondent reported the ‘child day’^2^ did not happen because the funding did not arrive on time. Others complained of widespread effects on health service provision, including maintenance of ambulances, and transportation of essential supplies to providers due to a lack of fuel.

*The consequences are we cannot feed the patient, we cannot order drugs in time, we cannot pay our suppliers in time, we cannot pay locum in time, we cannot maintain our vehicles, ambulances in time, so the consequences are so many and they really affect health care delivery in the district* — District level respondent

Insufficient funds resulted in the DHMT meeting to re-prioritize activities, which resulted in the budget process being less efficient and more top-down and less consultative than originally intended. Districts often acquired debts from suppliers, which affected relationships and sometimes ended up in court summons, with some DHMT members referring to themselves as ‘firefighter rather than an implementer’.

*Those delays affect our services […] we received a court summons from some suppliers, they are taking us to court because we have delayed paying them for the services rendered […]. Actually we have had water disconnected; the water board is always on our neck. […] even food, […] we are supposed to feed our patients but the suppliers sometimes refuse to give us food […] so instead of giving patients meat twice a week you may end up giving them beans throughout the week […]. —* District level respondent

District council officers sometimes agreed to re-allocate funds between sectors and to rely on partner funds, resulting in further dependency.

External funding via the SWAP or directly from donors often served to complement the DIP by supporting items or activities that were within the DIP and had no central level funding. A DHMT member reported if ‘what we included in the DIP has no resource[s] from the government, we lobby with the partners that are willing to support us in those interventions in our DIP’. District Health Officers reported that RMNCH was a high priority for donors and districts when developing budgets, comprising a large share of the health budget, although largely funded by donors.

*… if you talk about child, maternal health, for example the EPI*^3^*program, which is the main core of child health it takes about probably three tenths of the whole budget. —* District level respondent

*RMNCH as a program is getting more funding from implementing donors so* […] *we can say overall (…) it is better off as compared to other programs that have inadequate resources. —*District level respondent

There were concerns about the high degree of dependency on partner funds for the health sector at the district level, so that ‘the major investment comes from partners’ and district governments ‘provide the guidance and supervision’ (District level respondent). This resulted in concerns about the sustainability of funds going forward.

There were also concerns about the lack of coordination of partner funds, which may lead to skewed allocation of resources to areas favoured by the partners rather than those where there is the most need.

*Sometimes we feel like some partners the way they are coming in to support health, they pump in a lot of money in one program but if you look at the needs of the district, […] you can say if these other resources can be used in other programs that could be better. —* District level respondent

Respondents discussed the variety of activities that are funded by different funding sources. Although a large part of external funding was used to fund ‘training, orientation, refresher courses [and] maternal death audits’ (District level respondent), other recurrent transactions funds from government were used to keep health services running, such as ‘fuel, food for patients, water bills, sometimes the drugs’ (District level respondent).

## Discussion

This article presents the results of an in-depth analysis of health financing in Malawi at the sub-national level for all 26 districts in the years 2006 and 2011. District level analysis showed that per capita spending from all sources varied substantially across districts and that funding levels increased considerably between 2006 and 2011 in real terms, due to substantial growth in external and domestic funding levels. Districts in Malawi are heavily dependent on external funding for health, accounting for over 70% of funds in both years considered, which was of concern to government stakeholders. Domestic funding levels increased 6-fold over the period, with increases noted in all districts. However, domestic funds only accounted for 19% of total health expenditure in 2011. OOP funding levels were found to remain relatively constant over time, with OOP reducing slightly as a share of total health expenditure over the period of study. All funding sources were concentrated among wealthier districts (pro-rich), with the concentration being greatest for OOP, followed by domestic expenditure. External funding was the least pro-rich of the three sources, and moved towards being proportional in 2011. A previous study of aid allocations at the traditional authority level in Malawi found that poorer traditional authorities were more likely to receive aid (Marty *et al.* 2017). Although external funding was found to be complementary to government funds, local government representatives reported external funds were not allocated in an efficient manner at the district level. The high reliance on donor funding at district level is concerning, as such funding is known to be volatile (Celasun and Walliser 2008) (Martinez-Alvarez *et al.* 2017) affecting recipient country ability to plan. Consideration of methods for increasing domestic resource generation is needed through earmarked taxes for health, increased efficiency in tax revenue collection and more effective lobbying for funds to health (Meheus and McIntyre 2017; Mcintyre *et al.* 2017). The pro-rich distribution of funding is also concerning, and greater attention should be given to equity in the allocation of funding sub-nationally.

Higher levels of domestic and external funding were associated with lower levels of post-neonatal mortality - the group which saw the greatest decline in mortality; but there was no association between changes in financing and outcomes over time, possibly because of the short time period considered. A previous study also reported that infant mortality levels were significantly lower in areas where external funding was higher (Dionne *et al.* 2013); and a study using the same Aiddata dataset found that traditional authorities with higher prevalence of malaria were less likely to receive aid (Marty *et al.* 2017) though those that received some aid had lower prevalence than those that received none. Another study showed that higher levels of public health expenditure were associated with substantially lower levels of infant and under five mortality (Gani 2009). OOP payments were associated with higher levels of post-neonatal mortality, but this effect disappeared over time. OOP payments have been shown to be associated with lower access to care (Wang *et al*. 2015), which would lead to worse outcomes. It is also possible that households in districts with worse outcomes incur more expense, as a result of greater need for care. The qualitative data also highlighted the heavy reliance on external funding at district level, and concerns about the sustainability of donor funds in the longer term. Reliance on donor funding was partly adopted as a coping strategy to deal with delays in the receipt of domestic funding, which was considered insufficient to meet needs. External project funding was usually earmarked by districts for specific activities (e.g. training) or areas of joint donor and government priority (RMNCH services), suggesting coordination between donors and government for these funds. Prioritization of donor funding for specific activities and conditions that were underfunded is a rational response that has been reported elsewhere e.g. (Mayhew 2003). Other studies have highlighted the risks of reliance on donor funding pushing funding decisions away from local communities, with resource allocation and funding decisions being driven by donors (Jenniskens *et al.* 2012), and the potential misalignment of priorities (Stierman *et al.* 2013). We found evidence that districts in Malawi had some influence over how donor funding was used, and that external funding was allocated more equitably than other financing sources at district level. Misalignment of priorities is more likely to occur with vertical rather than sector wide support, and explains the preference of district managers for the latter form of funding. Although increasing levels of external funds is positive, one concern is that these displace domestic funds. Although we found that domestic funding levels were lower than that of external funding, we did not find evidence of a reduction in domestic funding in response to increased external funding, though some general budget support may have been captured in our estimate of domestic funding. Global resource tracking studies have found that countries with higher levels of external assistance tend to have lower levels of domestic funding (Lu *et al.* 2010), with some evidence pointing to the fungibility of development assistance for health at country level elsewhere (Martínez Álvarez *et al*. 2016).

Other district coping strategies to deal with delayed and insufficient domestic resources included dropping activities, or transferring funds across sectors, similar to that reported in Tanzania (Frumence *et al.* 2014). Although communities are engaged with district managers in the development of health plans, decisions to re-allocate funds across sectors or to cancel or modify planned activities were usually made by district stakeholders without lower level consultation. The challenge of securing funding to implement activities was especially great in poorer districts, as domestic funding was more highly concentrated in wealthier districts. The concentration of domestic funding in wealthier districts may partly stem from the decentralized nature of health financing in Malawi. A study in Indonesia also found that the introduction of financial decentralization benefitted wealthier districts more than poorer districts (Abdullah and Stoelwinder 2007).

This study is subject to some methodological limitations. First, we present our analysis in relation to 2 years (2006 and 2011), however, in practice the period of focus for each data source was not entirely consistent, with domestic funding reflecting the financial year 2005/06 and 2010/11, and OOP funding reflecting the calendar years 2005 and 2010, and mortality estimates derived from 2006 and 2010 sources. However, we do not expect major year on year variation in funding levels or outcomes. We estimated domestic expenditure from budget books ‘approved budget’ figures, rather than using actual expenditure, as these were considered the most reliable source of financing data at the time of study. However, our measure of domestic funding may include donor funding that is channelled through the MoF as general budget support, as there was no mechanism for identifying and removing such funds from our estimates; it also includes locally generated funds. Therefore, although we report an increase in domestic funding over time, this may partly be a reflection of the consolidation of general budget support which includes external funding, in addition to increased domestically sourced revenue. Further, the approved budget figures are generated half way through the financial year so any major deviations in the second half of the year from what was budgeted will not be captured. At the district level we estimated external expenditure by allocating an equal expenditure to each year for each project, as annual disbursement information was not available. However, there will likely be some disparity between our estimates and actual funds received in a given year. Further, external funding captured within AidData represents just over 80% of all aid, so actual external funding levels will be higher than that reported. Although there is a potential risk of double counting general budget support within our domestic and external funding categories, in practice, we did not find any general budget support funding within those disbursements which had been assigned to the health sector by the MoF, suggesting this was not an issue. The geocoded AidData database for Malawi is sanctioned by the MoF, Economic Planning and Development and includes data from nearly 30 donors. We believe this is the most accurate estimate available of external expenditure in Malawi at the sub-national level. When analysing associations between financing and outcomes, we only explored associations between financing and outcome variables at a particular point in time, and we did not control for other factors which may have affected outcomes, nor did we account for lagged effects. Further, our analysis is aggregated at the district level, and we examined associations between general health aid and child mortality. Associations between funding to child health and child mortality may have been different, but it was not possible to disaggregate domestic and OOP payments by health conditions or population groups.

Finally, the qualitative analysis is also subject to limitations. First, respondents may not have felt comfortable being recorded and may not have wanted to share some of their personal views on sensitive topics of health financial management. Second, although the coding tree was developed by two researchers, the rest of the coding and analysis was undertaken by one researcher, which may have influenced the results presented here. We were also unable to interview development partners to obtain their perspective on the allocation of external funding in Malawi nor to interviews patients or households to understand their perceptions of OOP payments and changes over time.

In order to improve the availability of domestic funding for health, stronger public financial management practices are needed to ensure the timely availability of adequate levels of funding and to respect local level accountability in planning for how funds will be used. Although the ongoing reliance on donor funding is essential in the short term, pooled mechanisms have clear advantages for local planners, due to the greater autonomy in allocating funds to address local priorities, and these should be allocated directly to districts to minimize delays in the receipt of funds. Although pooled funding was frozen during the cash gate scandal, such mechanisms should be encouraged going forward. Given the increased decision making power of districts, donors might also consider allocating funds directly to districts to avoid delays associated with transfers through the MoF.

Although generating additional domestic resources is clearly a key priority to meet the resource gap needed to achieve and make progress towards universal health coverage, governments also need to keep a close eye on how these resources are allocated sub-nationally to maximize equity and ensure allocations are commensurate to health need. To do so will require investment in resource tracking tools to facilitate consistent and reliable measurement of funding at the sub national level for consistent years and time frames, and also tracking funding by beneficiary group. WHO’s systems of health accounts methodology which has been employed in a number of countries is a promising move in this direction together with AidData’s geocoded datasets which offer the opportunity to explore external funding sub-nationally which is not currently possible with the OECD’s creditor reporting system. However, more disaggregated reporting of such funds within AidData’s database would be helpful to more precisely determine annual expenditures.

## Funding

This work was funded through a sub-grant from the U.S. Fund for UNICEF under their Countdown to 2015 for Maternal, Newborn and Child Survival Grant (no. OPP1058954) from the Bill and Melinda Gates Foundation.

*Conflict of interest statement*. None declared.

## Footnotes

1 Although general budget support (including SWAP) was suspended in Malawi from 2014 following a series of corruption scandals known as ‘cash gate’; at the time of the research donor funds were channelled as outlined here.

2 Child Health Days take place in the whole of Malawi and involve the delivery of a package of interventions, including exclusive breastfeeding, feeding a sick child, vitamin A rich foods, de-worming, hand washing with soap, use of iodized salt and insecticide treated nets (http://www.unicef.org/esaro/5479_child_health_days.html)

3 EPI, extended program for immunization.
